# Pleistocene divergence in the absence of gene flow among populations of a viviparous reptile with intraspecific variation in sex determination

**DOI:** 10.1002/ece3.7458

**Published:** 2021-03-25

**Authors:** Peta Hill, Erik Wapstra, Tariq Ezaz, Christopher P. Burridge

**Affiliations:** ^1^ Discipline of Biological Sciences University of Tasmania Sandy Bay Tas. Australia; ^2^ Institute for Applied Ecology University of Canberra Bruce ACT Australia

**Keywords:** genetic sex determination, GSD, *Niveoscincus*, sex chromosome, temperature‐dependent sex determination, TSD

## Abstract

Polymorphisms can lead to genetic isolation if there is differential mating success among conspecifics divergent for a trait. Polymorphism for sex‐determining system may fall into this category, given strong selection for the production of viable males and females and the low success of heterogametic hybrids when sex chromosomes differ (Haldane's rule). Here we investigated whether populations exhibiting polymorphism for sex determination are genetically isolated, using the viviparous snow skink *Carinascincus ocellatus*. While a comparatively high elevation population has genotypic sex determination, in a lower elevation population there is an additional temperature component to sex determination. Based on 11,107 SNP markers, these populations appear genetically isolated. “Isolation with Migration” analysis also suggests these populations diverged in the absence of gene flow, across a period encompassing multiple Pleistocene glaciations and likely greater geographic proximity of populations. However, further experiments are required to establish whether genetic isolation may be a cause or consequence of differences in sex determination. Given the influence of temperature on sex in one lineage, we also discuss the implications for the persistence of this polymorphism under climate change.

## Graphical abstract

Polymorphism for sex determining system might be expected to isolate gene pools, given strong selection for the production of viable males and females. We investigated this question using a rare example of a species exhibiting polymorphism for sex determination: the viviparous snow skink *Carinascincus ocellatus*.

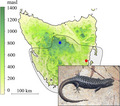

## 1 INTRODUCTION

Speciation occurs when lineages become reproductively isolated due to a trait polymorphism. If mating success is lower among individuals that differ for a given trait, lineages will diverge in the frequency of that trait and experience further reductions in gene flow, potentially initiating speciation. Trait polymorphisms that may particularly promote speciation include those related to mate choice (e.g., sexual dichromatism; Jenck et al., 2020; Portik et al., 2019), breeding phenology (Taylor & Friesen, 2017), parity (Horreo et al., 2019), vocalizations (Campbell et al., 2019; Luo et al., 2017), and karyotype, particularly those involving chromosomes that determine sex (i.e., sex chromosomes; Bracewell et al., 2017; Faria & Navarro, 2010; Kitano et al., 2009; O'Neill & O'Neill, 2018; Zhang et al., 2015).

Sex determination, which directs gonadal differentiation in sexually reproducing organisms (Bachtrog et al., 2014; Hayes, 1998), often has a strong chromosomal basis which is highly conserved within groups, reflecting strong selective constraint on the production of viable males and females. For example, the systems of genetic sex determination (GSD) are fixed in therian mammals and birds, represented by heterogametic XY male and ZW female chromosome systems, respectively (Graves, 2006; Ohno, 1967). However, sex determination is comparatively labile in reptiles (Alam et al., 2018; Janzen & Phillips, 2006; Johnson Pokorna & Kratochvil, 2016), where offspring sex is controlled either by genes (both male and female heterogametic systems), the environment (e.g., temperature dependant sex determination, TSD), or by a combination of genes and the environment (GSD with environmental effects, GSD + EE; Cornejo‐Paramo et al., 2020; Ezaz et al., 2006; Holleley et al., 2015; Quinn et al., 2007; Radder et al., 2008; Sarre et al., 2004). Although transitions among these systems were initiated as an intraspecific polymorphism, it is unknown whether they were accompanied by genetic isolation. Within squamates, the family Scincidae shows evidence of conserved sex chromosomal regions between some lineages (Cornejo‐Paramo et al., 2020; Dissanayake et al., 2020) in addition to temperature sensitivity in sex determination (Holleley et al., 2016). However, variation in the degree of sex chromosome differentiation, number of sex chromosomes (Ezaz et al., 2009), and system of heterogamety (Patawang et al., 2018) exists, and our understanding of the mechanisms of evolutionary transitions in sex determination and how they impact demographics remains poor.

Low mating success is expected among individuals when differences in sex determination reflect gross chromosomal differences (e.g., sex chromosome presence, composition, or system of heterogamety; Haldane, 1922; Lima, 2014; O'Neill & O'Neill, 2018; Phillips & Edmands, 2012). However, not all changes to the sex chromosomes result in incompatibilities. When genes and temperature interact to determine sex, a temperature override of the genetic sex determination signal can produce individuals whose sexual phenotype does not match their sexual genotype (known as sex reversal). This phenomenon is occurring in wild populations of the central bearded dragon, *Pogona vitticeps*, which has a thermosensitive ZW/ZZ system of sex determination, resulting in the production of females (normally ZW) with a male genotype (ZZ) (Holleley et al., 2015). While the W chromosome has been lost in these sex‐reversed females, and the thermal threshold for sex reversal is evolving in this system (Holleley et al., 2015; Quinn et al., 2011), the Z chromosomes remain homologous and ZZ males can successfully breed with ZZ females under laboratory conditions (Holleley et al., 2015). For a transition in sex‐determining system to lead to postzygotic incompatibilities via hybrid inviability or sterility, the transition must involve changes to the sex chromosomes such that they show deleterious interactions on a hybrid background (Haldane, 1922).

The viviparous Tasmanian spotted snow skink, *Carinascincus ocellatus* (formerly *Niveoscincus*), is an extraordinary example of a species exhibiting incipient divergence in sex determination (Cunningham et al., 2017; Pen et al., 2010; Wapstra et al., 2004). This species is widely distributed across a broad altitudinal and climatic range in Tasmania, from sea level to 1,200 m (Wapstra & Swain, 2001; Wapstra et al., 1999). Long‐term field data and laboratory experiments document variation in sex ratio with temperature at a comparatively warm, low elevation population, but parity of sex ratios regardless of temperature at a cool high elevation population (Pen et al., 2010; Wapstra et al., 2009). In addition, population‐specific sex‐linked DNA variation exists in both *C. ocellatus* sex‐determining systems and sex chromosomes in the two populations have minor structural differences (Hill et al., 2021). Therefore, a high elevation population exhibits GSD (50:50 sex ratios facilitated by high elevation XY sex chromosomes in the absence of thermosensitivity), while a low elevation population has GSD + EE (biased sex ratios facilitated by low elevation XY sex chromosomes with thermosensitivity; Hill et al., 2018). In the GSD + EE population, warmer years result in a female‐biased sex ratio; cooler years result in a male bias (Cunningham et al., 2017; Pen et al., 2010). In *C. ocellatus*, the divergence in sex determination appears driven by climate‐specific selection: early birth confers a fitness advantage to females at low elevation because birth date influences the onset of maturity and females have a higher lifetime reproductive fitness when born early (Pen et al., 2010). At high elevations, the shorter reproductive season and longer period between birth and maturation preclude any advantage for either sex based on birth date (Pen et al., 2010). In addition, interannual weather fluctuations selects against GSD + EE at high altitudes to prevent extreme sex ratios (Pen et al., 2010). *C*. *ocellatus* populations would have experienced this climate‐specific selection as they dispersed from refugia during the interglacial periods of the Pleistocene.

Although several studies have provided information regarding the genetic isolation of GSD and GSD + EE *C. ocellatus* populations, they each contain caveats (Cliff et al., 2015; Hill et al., 2018). Firstly, phylogeographic analysis of mitochondrial DNA (mtDNA) revealed a lack of reciprocal monophyly between these populations and suggested that the ancestors of the GSD and GSD + EE lineages likely occupied shared lowland refugia during Pleistocene glaciations, including the last glacial maximum, and were potentially interbreeding (Cliff et al., 2015). Furthermore, the species is presently more‐or‐less continuously distributed between the GSD and GSD + EE sites, with no obvious large‐scale barriers to movement (Cliff et al., 2015), suggesting the possibility of contemporary gene flow. However, the lack of mtDNA genetic structuring among these populations may not refute contemporary genetic isolation of these sex‐determining systems, given the potential for mitochondrial incomplete lineage sorting to persist in large and recently diverged populations (Funk & Omland, 2003). Secondly, a detailed genomic analysis identified loci with population‐specific sex‐linked variation (33 loci in the GSD and 42 loci in the GSD + EE populations; Hill et al., 2018), suggesting genetic isolation. Similarly, linkage disequilibrium among sex‐linked SNPs common to both populations is greater in the GSD than GSD + EE population (Hill et al., 2018). This suggests disparate inhibition of sex chromosome recombination (and differentiation) among populations, despite some regions being conserved relative to other taxa (Cornejo‐Paramo et al., 2020). However, crosses between individuals with different sex chromosomes could still maintain population‐specific sex‐linked loci, while homogenizing autosomal variation depending on the strength of selection on hybrid incompatibilities (Presgraves, 2018). Thirdly, while we have attempted to cross‐breed these populations, and copulations occurred (suggesting no strong premating isolation), there were no subsequent births, but breeding experiments involving within‐population crosses also had low success. Therefore, we lack knowledge of whether the divergence of these sex‐determining systems may have impacted autosomal gene flow between their populations more generally (without precluding genetic isolation by other mechanisms).

Here we used “Isolation with Migration” models (Hey & Nielsen, 2004) and neutral, nonsex‐linked single nucleotide polymorphisms (SNPs) to investigate whether autosomal gene flow has accompanied divergence of the GSD and GSD + EE populations of *C*. *ocellatus*. This approach is widely applicable to the exploration of whether gene flow between lineages has been disrupted by divergence in traits (Hey, 2010; Hey et al., 2018; Hey & Nielsen, 2004; Runemark et al., 2012). Furthermore, we used this approach to estimate the age of the divergence of GSD and GSD + EE lineages, to address whether gene flow occurred between them during their divergence. Reptiles' close link with the thermal environment makes them a compelling group for understanding the interactions between climate‐mediated natural selection on sex‐determining systems and gene flow among lineages. We discuss our findings in the context of sex determination transitions against a background of Pleistocene climate fluctuations and infer the consequences to GSD and GSD + EE populations of *C*. *ocellatus* under climate change.

## 2 METHODS

### 2.1 Study populations

**FIGURE 1:**
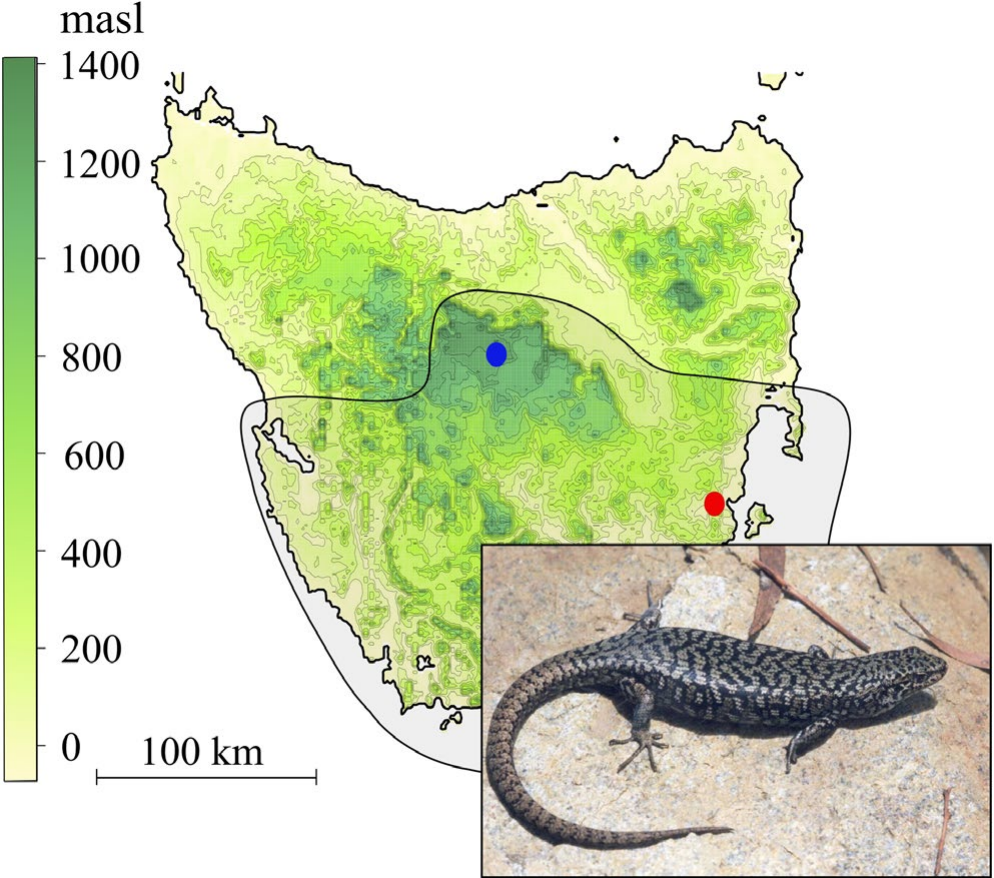
Locations of GSD (high elevation; blue circle) and GSD + EE (low elevation; red circle) populations of *Carinascincus ocellatus* with divergent sex determining systems. Altitudinal gradient is indicated, and grey shaded region represents the mitochondrial clade to which these populations belong (Cliff et al., 2015). Inset: *C. ocellatus*

We studied populations of *C. ocellatus* representing the climatic extremes of this species' range: a warmer low elevation population (42 34′S, 147 52′E; elevation 50 m) and a cooler high elevation population (41 51′S, 146 34′E; elevation 1,200 m. Figure 1). These are the same populations that underpinned research on sex determination and sex‐linked DNA sequences in this species (Cunningham et al., 2017; Hill et al., 2018; Wapstra et al., 2004, 2009). Mitochondrial genotypes for five and four individuals were included representing the GSD and GSD + EE populations, respectively, and likewise 42 and 44 individuals for nuclear SNPs.

### 2.2 Neutral autosomal SNP and mitochondrial markers

The concatenated mitochondrial sequences (NADH2 and NADH4) were obtained from Cliff et al., (2015). Neutral autosomal SNPs were derived from the dataset of Hill et al., (2018), obtained using a high‐throughput double digest, restriction enzyme reduced representation sequencing approach (Kilian et al., 2012). All sex‐linked SNPs from this dataset were removed for this analysis. Secondaries (additional SNPs on the same fragment) were removed from remaining loci using custom R script (R Core Team, 2017); the SNP with the highest reproducibility and polymorphic information content from each locus was retained. SNP genotypes with an average reproducibility < 0.5, a call rate of <0.9 and loci monomorphic within populations were also removed using the dartR package (Gruber & Georges, 2019) in R. This left 11,107 SNPs with an average reproducibility of 0.99 and call rate of 0.98. These SNPs were used to calculate pairwise Fst, visualize the genetic similarity of the populations via a principal coordinates analysis in the dartR package (Gruber & Georges, 2019), and to identify individuals of mixed origin using STRUCTURE v 2.3.4 (Pritchard et al., 2000). We used the admixture model as implemented in STRUCTURE with no prior information on geographic origin included. Runs were replicated five times, and we assessed the likelihood values for *K* = 1–5 using the Evanno method (Evanno et al., 2005) implemented in STRUCTURE HARVESTER (Earl & vonHoldt, 2012). For each run, we used a burnin of 10^5^ iterations and a further run length of 10^6^ iterations. SNPs putatively under selection (Fst in the 5th percentile) and those not in Hardy–Weinberg equilibrium (HWE; *p* < .05) in either population were then filtered from the data using Genepop (Rousset, 2008). From the remaining 9,453 loci, a set of 100 SNPs were chosen at random for coalescent analysis; linkage among these loci was ruled out (*R* ^2^ < 0.5) using the “*genetics”* package (Warnes et al., 2013) in R.

### 2.3 Isolation with Migration analysis

**FIGURE 2:**
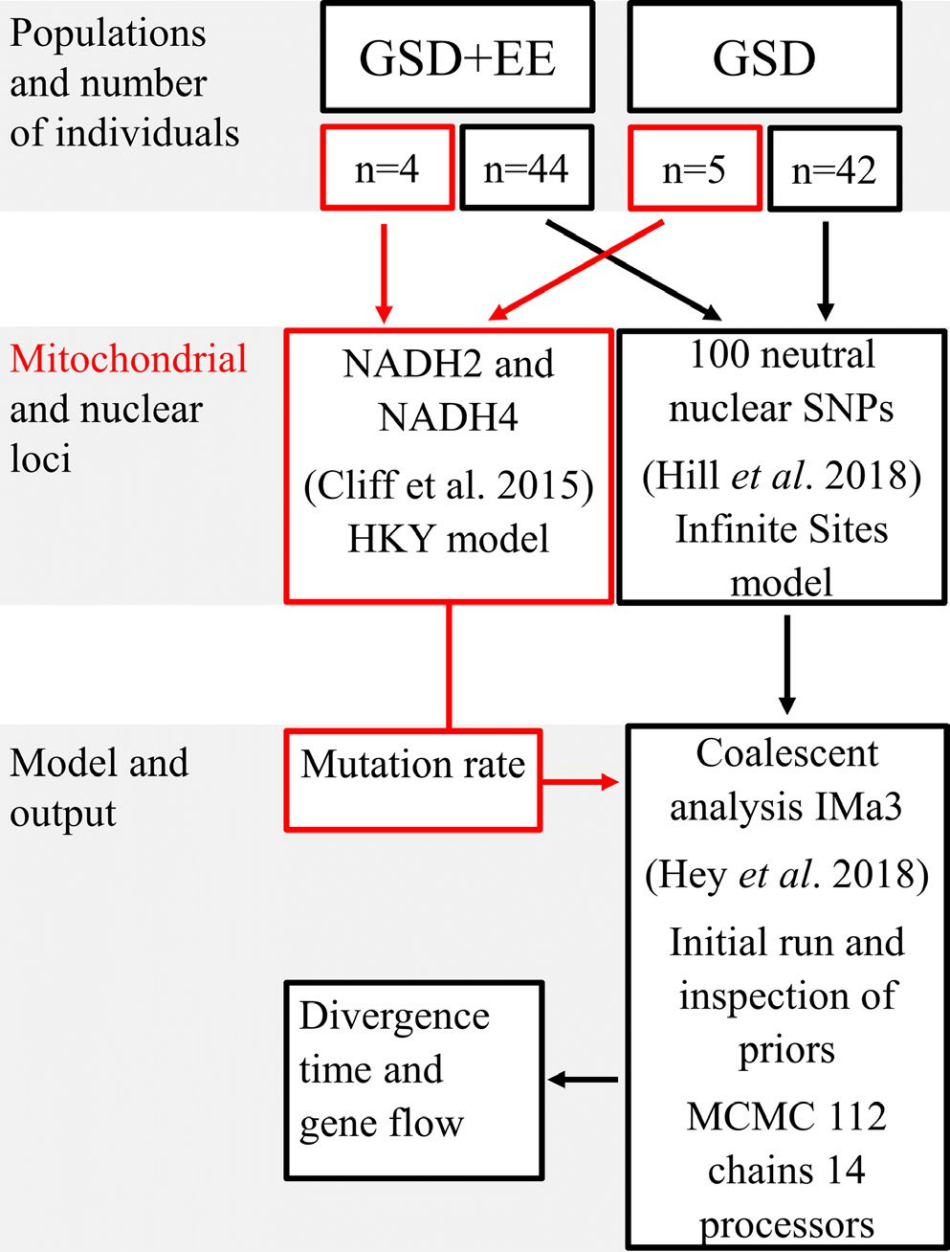
Flowchart for estimating divergence time and gene flow amongst GSD (high elevation) and GSD + EE (low elevation) populations of *Carinascincus ocellatus* using an Isolation with Migration (IMa3) framework. Red denotes steps specific to the mitochondrial locus

The level of gene flow accompanying divergence of GSD and GSD + EE populations, along with their duration of divergence, was assessed under the “Isolation with Migration” Bayesian framework of Hey and Nielsen (2004), employing IMa3 (Hey et al., 2018). Mitochondrial sequences and dart‐tags containing the neutral nuclear SNPs were analyzed concurrently to estimate lineage‐splitting time and rates of gene flow between lineages in each direction (Figure 2). The HKY mutation model (Hasegawa et al., 1985) was employed for mtDNA sequence data, while the infinite sites model (Kimura, 1969) was employed for nuclear SNPs (Hey & Nielsen, 2004). Uniform priors were employed for divergence time and population size parameters, while exponential priors were employed for gene flow, given an expectation that low rates were likely (mean of prior distribution 6 × 10^−06^, approximating one individual per generation). Upper limits on uniform priors were initially set broadly, and then based on inspection of posterior distributions, were narrowed in a subsequent run to encompass the range of this posterior plus a margin of error; overly large priors reduce the precision of estimates given the use of a finite number of bins to represent the posterior distribution.

Isolation with Migration analysis was performed using Markov Chain Monte Carlo sampling with 112 chains distributed across 14 processors, and a geometric chain heating scheme with first and second heating parameters of 0.95 and 0.50, respectively. To reduce overall run‐time, an initial analysis was run and traces inspected to ensure stationarity of the sampling distribution was achieved, and this was then used to seed four simultaneous analyses, each run for 24 hr following a 10‐min burnin and using unique random number seeds, to ensure independence among runs. All runs were assessed for convergence using Tracer 1.7.1 (Rambaut et al., 2018) prior to combining the results. In total, 111,643 genealogies were retained for estimation of model parameters.

Information on mutation rate was employed to scale output into units of years (divergence time, gene flow). Mitochondrial mutation rates were employed in the analysis, against which mutation rates at the nuclear loci would be scaled. We followed the mean rate estimate of 1.52% divergence per million years based on calibrations from other squamates (Chapple et al., 2011) and used by Cliff et al., (2015). To account for potential variation in mutation rate (Ansari et al., 2019; Ho et al., 2005, 2007), we explored the consequences of using a faster rate of 2.3% divergence per million years, reported from Canary Islands skinks (Brown & Pestano, 1998). Faster rates may be more applicable to reconstructing demographic history over recent (<100,000 yr) timescales (Burridge et al., 2008). Failure to entertain time‐dependent rates of molecular change will lead to overestimation of divergence time and underestimation of gene flow (Burridge et al., 2008).

## 3 RESULTS

**FIGURE 3:**
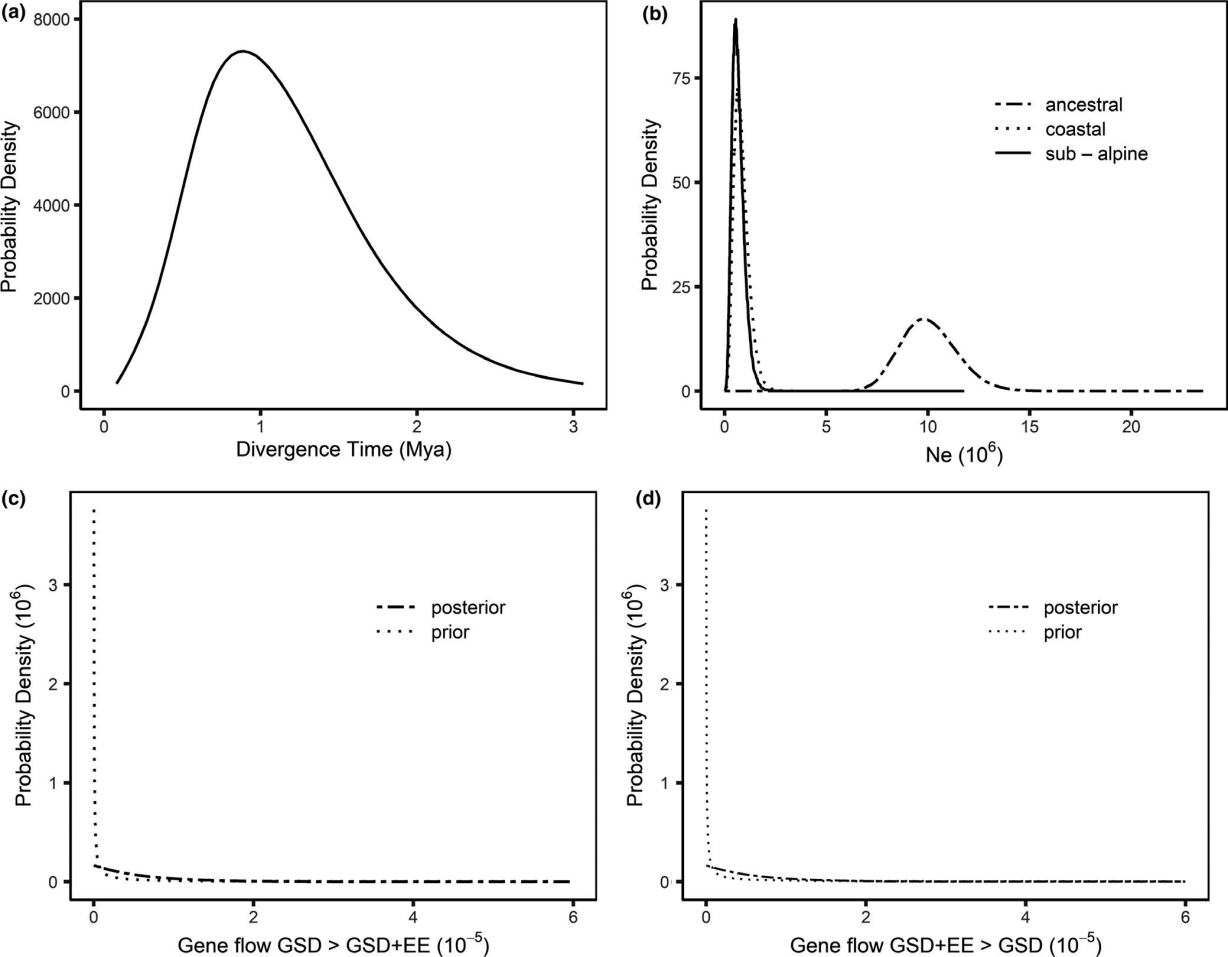
Estimated marginal posterior densities for parameters from the Isolation with Migration (IMa3) analysis of *Carinascincus ocellatus* populations with divergent sex determining systems. (a) divergence time of populations, (b) Effective population sizes (Ne) of ancestral, GSD and GSD + EE populations and gene flow from (c) GSD to GSD + EE and (d) GSD + EE to GSD populations

The divergence of GSD + EE (low elevation) and GSD (high elevation) populations of *C. ocellatus* occurred under negligible gene flow and commenced between 0.61 and 0.92 Mya (highest posterior density 0.16–2.30 Mya under different mutation rates, Table 1; Figure 3). While analysis with a faster mutation rate (2.3% divergence/Myr) produced a more recent estimate of the divergence time (Table 1), divergence still occurred within the Pleistocene and substantially predated the last glacial maximum. This result, and that of Cliff et al., (2015), indicates that GSD and GSD + EE lineages were likely sympatric, and definitely more proximate, at low elevation refugia through multiple Pleistocene glaciations, yet they still diverged under negligible gene flow.

**TABLE 1:** Posterior estimates of divergence (split) time, gene flow and effective population size (Ne) of GSD and GSD+EE populations of *Carinascincus ocellatus* based on mitochondrial mutation rates of 1.52% and 2.30% divergence per million years. Median values from posteriors are reported, along with 95% highest posterior densities (HPD) for population splitting time (values for migration posterior were sensitive to prior distribution, and hence are not reported). Note that migration rate (gene flow) posteriors are described “backwards in time”

Mutation rate (% per Myr)	Split time (Mya)	95% HPD interval	Gene flow (per gene per year)		Population size (Ne, million individuals)		
			GSD + EE to GSD	GSD to GSD + EE	GSD + EE	GSD	Ancestral
1.52	0.92	0.24–2.30	3 × 10^−8^	4 × 10^−8^	0.63	0.55	9.8
2.30	0.61	0.16–1.50	3 × 10^−8^	4 × 10^−8^	0.42	0.36	6.5

**FIGURE 4:**
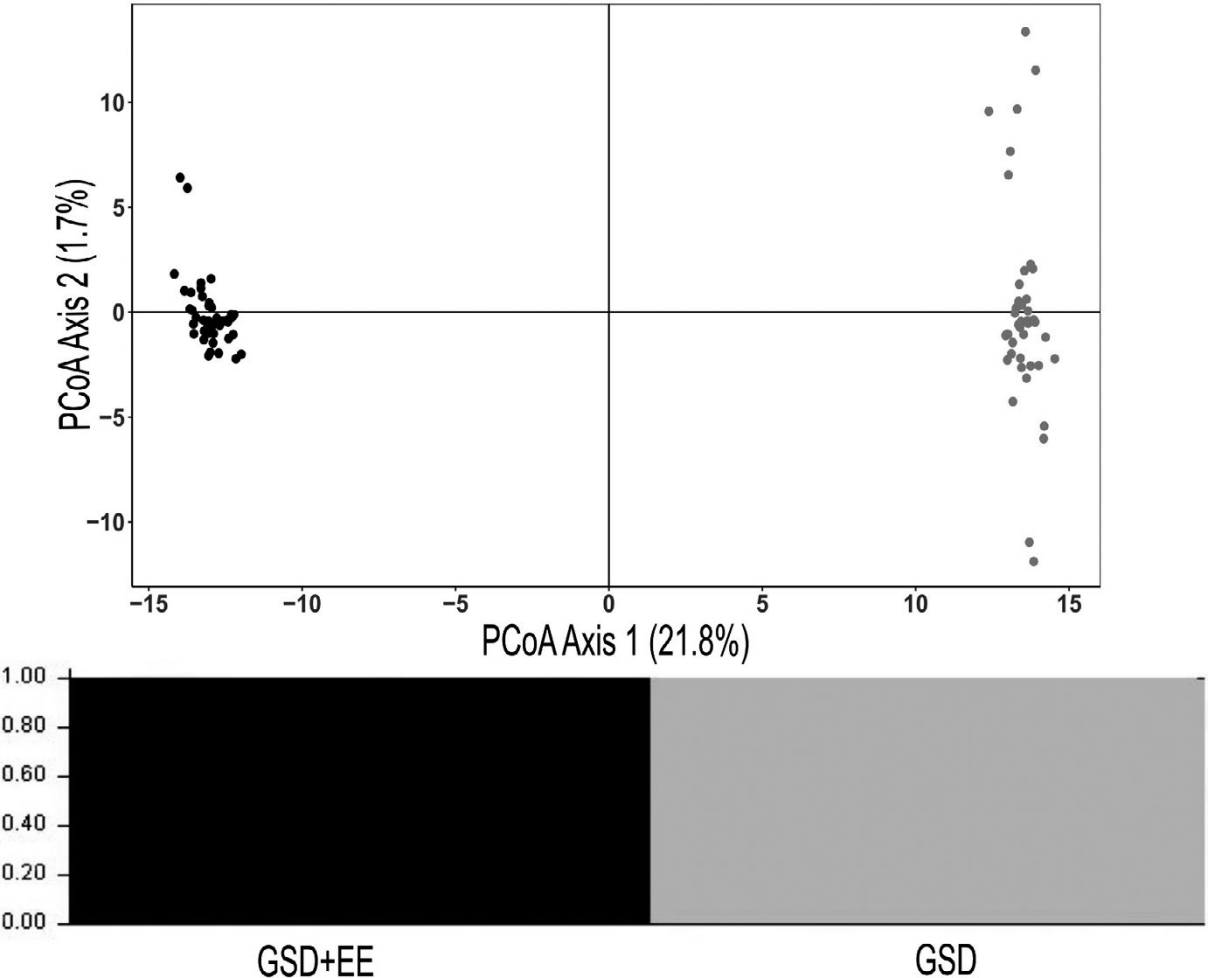
Principle Coordinates Analysis (upper panel) and STRUCTURE (lower panel) analysis conducted on 11,107 SNP genotypes of individuals collected from two populations of *Carinascincus ocellatus* with divergent sex determining systems. Populations are GSD (grey) and GSD + EE (black)

Pairwise Fst between the populations is 0.24, consistent with negligible gene flow compared with both inter and intraspecific values reported for squamates (Dennison et al., 2012; Koc et al., 2017; Tucker et al., 2014). In the principal coordinates analysis, the major axis of variation, PC1, explained 21.8% of the total variation in SNP genotypes and placed individuals into two distinct groups representing our populations, with PC2 explaining a further 1.7% (Figure 4). These results were corroborated by STRUCTURE which assigned all individuals as pure GSD or pure GSD + EE in origin (*K* = 2; Figure 4).

## 4 DISCUSSION

The presence of contemporary genetic structure and an absence of gene flow during the Pleistocene divergence of GSD and GSD + EE populations of *C. ocellatus*, across recurrent periods of likely sympatry, raises the possibility that divergence in sex‐determining system promoted broader genetic isolation. When mutations occur on a sex chromosome that disrupt sex determination and lead to sex ratio skews (e.g., hybrid sterility in the heterogametic sex which leads to sex ratios in favor of the homogametic sex; Haldane's rule, e.g., Olsson et al., 2004), compensatory mutations that return sex ratios to parity are favored (O'Neill & O'Neill, 2018). These can occur on the alternative sex chromosome or the autosomes (Meiklejohn & Tao, 2010). This process, driven by genetic conflict over the sex ratio, presents opportunities for further divergence of sex determination. If subsequent mating occurs between lineages diverging in sex determination, incompatibilities at the genomic or chromosomal level can result in postzygotic isolation, inhibiting gene flow (Faria & Navarro, 2010; Meiklejohn & Tao, 2010; Zhang et al., 2015). The Pleistocene also represents an important period of speciation in ectotherms (Avise et al., 1998). A mutation, or a polymorphism for epigenetic regulation, arising and segregating on sex chromosomes in the ancestral *C. ocellatus* population, once exposed to selection gradients across climate during Pleistocene glacial cycles, could rapidly inhibit gene flow between sex‐determining systems. Any of the population‐specific sex‐linked loci described for *C. ocellatus* (Hill et al., 2018) could be responsible for initiating the observed differences in sex determination, and potentially, the isolation of their populations.

Although we have observed low gene flow during the divergence of sex‐determining systems in *C. ocellatus*, we cannot yet exclude the possibility that divergence in sex determination postdates the emergence of an alternative isolating trait. For instance, the impact of geographic distance on genetic isolation requires consideration; in essence, whether the genetic isolation we observe here exceeds that across a comparable geographic scale within a sex‐determining system. Furthermore, testing whether sex reversal contributes to observed population‐specific sex determination in *C. ocellatus* is important, as sex‐reversed individuals can provide a conduit for gene flow between systems (Holleley et al., 2015). While morphologically distinct sex chromosomes are known to isolate lineages (Phillips & Edmands, 2012), the degree of differentiation required for this to occur is unknown. In the case of *C. ocellatus*, sex chromosomes are similar between systems, with slightly more repeats and heterochromatin on Y chromosomes from the GSD population (Hill et al., 2021). Likewise, the number of population‐specific sex‐linked markers (GSD *n* = 33, GSD + EE *n* = 42) is small relative to those still shared between populations (*n* = 206; Hill et al., 2018). High chromosomal similarity would also be expected if the difference in sex‐determining system is merely a polymorphism for a temperature threshold in a shared gene product (Quinn et al., 2011). On the other hand, close examination of the life history of this species has not revealed strong evidence for population‐specific local adaptation in traits that may explain their genetic isolation (Cadby et al., 2014; Caldwell et al., 2017; Cliff et al., 2015; Wapstra & Swain, 2001; Wapstra et al., 1999). For example, temperature reaction norms for gestation length and offspring development are remarkably similar in each population (Cunningham et al., 2020), however, further studies will reveal if local thermal adaptation has occurred since isolation resulting in population‐specific thermosensitivity of sex determination. Regardless, whether sex determination isolated populations or occurred subsequent to their isolation, our estimate of divergence time places a lower limit on the timeframe of their divergence.

With evidence for isolation of populations at the extremes of the species range, it is important to understand how different sex‐determining systems will impact population responses to increases and fluctuations in temperature over rapid timescales. As temperatures rise, sex ratios across the current *C. ocellatus* range will become increasingly female biased in populations with GSD + EE (Cunningham et al., 2017; Pen et al., 2010; Wapstra et al., 2009). Climate change is also shifting species' distributions (Bonebrake et al., 2018), with higher elevations becoming accessible to phenotypes that were historically excluded (Sinervo et al., 2010). Increased population growth due to an excess of females in GSD + EE populations (Wedekind, 2002), will also promote range shifts (Boyle et al., 2014, 2016). If the GSD and GSD + EE sex‐determining systems isolate these populations, this will inhibit the transmission of potentially beneficial autosomal alleles between populations on secondary contact, representing a potential impediment to their adaptation to changing environmental conditions. Alternatively, as climates warm and temperature fluctuations become more extreme, and if dispersal is a limiting factor, a mismatch may occur between the climate experienced by populations and their ability to sustain fundamental metabolic processes, leading to local extinctions (Sinervo et al., 2010). Experiments designed to map the geographic distribution of sex‐determining systems in *C*. *ocellatus*, combined with modeling of future climate scenarios across its range, will confirm potential contact zones between alternative sex‐determining systems and regions of the current and future distribution where mismatches are most likely to occur between optimal sex determination and climate.

## CONFLICT OF INTEREST

The authors have no conflicts of interest to declare.

## AUTHOR CONTRIBUTIONS

**Peta Hill:** Conceptualization (equal); data curation (lead); formal analysis (equal); funding acquisition (equal); methodology (equal); project administration (equal); writing–original draft (lead). **Erik Wapstra:** Conceptualization (equal); funding acquisition (lead); project administration (lead); resources (equal); supervision (equal); writing–review and editing (equal). **Tariq Ezaz:** Conceptualization (equal); formal analysis (equal); funding acquisition (equal); project administration (equal); resources (equal); supervision (equal); writing–review and editing (equal). **Christopher Burridge:** Conceptualization (equal); formal analysis (equal); funding acquisition (equal); methodology (lead); project administration (equal); software (lead); supervision (lead); writing–review and editing (equal).

## Data Availability

The data used in this study have been deposited to Dryad https://doi.org/10.5061/dryad.2547d7wq1.
